# Spirometry test values can be estimated from a single chest radiograph

**DOI:** 10.3389/fmed.2024.1335958

**Published:** 2024-03-06

**Authors:** Akifumi Yoshida, Chiharu Kai, Hitoshi Futamura, Kunihiko Oochi, Satoshi Kondo, Ikumi Sato, Satoshi Kasai

**Affiliations:** ^1^Department of Radiological Technology, Faculty of Medical Technology, Niigata University of Health and Welfare, Niigata, Japan; ^2^Major in Health and Welfare, Graduate School of Niigata University of Health and Welfare, Niigata, Japan; ^3^Konica Minolta, Inc., Tokyo, Japan; ^4^Kyoto Industrial Health Association, Kyoto, Japan; ^5^Graduate School of Engineering, Muroran Institute of Technology, Muroran, Japan; ^6^Department of Nursing, Faculty of Nursing, Niigata University of Health and Welfare, Niigata, Japan

**Keywords:** pulmonary function test, chest radiography, artificial intelligence, spirometry, deep learning

## Abstract

**Introduction:**

Physical measurements of expiratory flow volume and speed can be obtained using spirometry. These measurements have been used for the diagnosis and risk assessment of chronic obstructive pulmonary disease and play a crucial role in delivering early care. However, spirometry is not performed frequently in routine clinical practice, thereby hindering the early detection of pulmonary function impairment. Chest radiographs (CXRs), though acquired frequently, are not used to measure pulmonary functional information. This study aimed to evaluate whether spirometry parameters can be estimated accurately from single frontal CXR without image findings using deep learning.

**Methods:**

Forced vital capacity (FVC), forced expiratory volume in 1 s (FEV_1_), and FEV_1_/FVC as spirometry measurements as well as the corresponding chest radiographs of 11,837 participants were used in this study. The data were randomly allocated to the training, validation, and evaluation datasets at an 8:1:1 ratio. A deep learning network was pretrained using ImageNet. The input and output information were CXRs and spirometry test values, respectively. The training and evaluation of the deep learning network were performed separately for each parameter. The mean absolute error rate (MAPE) and Pearson’s correlation coefficient (*r*) were used as the evaluation indices.

**Results:**

The MAPEs between the spirometry measurements and AI estimates for FVC, FEV_1_ and FEV_1_/FVC were 7.59% (*r* = 0.910), 9.06% (*r* = 0.879) and 5.21% (*r* = 0.522), respectively. A strong positive correlation was observed between the measured and predicted indices of FVC and FEV_1_. The average accuracy of >90% was obtained in each estimation of spirometry indices. Bland–Altman analysis revealed good agreement between the estimated and measured values for FVC and FEV_1_.

**Discussion:**

Frontal CXRs contain information related to pulmonary function, and AI estimation performed using frontal CXRs without image findings could accurately estimate spirometry values. The network proposed for estimating pulmonary function in this study could serve as a recommendation for performing spirometry or as an alternative method, suggesting its utility.

## 1 Introduction

Imaging tests and pulmonary function tests (PFTs) are two important examination modalities that are fundamental to respiratory medicine. Imaging tests are used to diagnose abnormalities based on the anatomy and morphology of the respiratory tract, whereas PFTs are used to evaluate the physiological functions of the respiratory tract as quantitative values. Spirometry is a relatively simple method for measuring the ventilatory performance and is performed in routine practice and as part of medical examinations. Spirometry quantitatively measures the pulmonary capacity and velocity by determining the pressure and flow rate. The results are interpreted based on the symptoms and other clinical findings. Forced vital capacity (FVC) and forced expiratory volume in 1 s (FEV_1_) can be measured using spirometry. These indices can be evaluated relative to the decline in pulmonary function by calculating the ratio of the measured values (% FVC and % FEV_1_) to the representative values corresponding to the individual’s age, height, and sex. Post-bronchodilator FEV_1_/FVC <0.7 indicates obstructive ventilatory defects and is used as a strong diagnostic criterion (1–4). Thus, FVC, FEV_1_ and FEV_1_/FVC are important clinical assessment indices (5, 6). They allow for earlier detection of diseases that affect pulmonary function, such as chronic obstructive pulmonary disease (COPD) and asthma, than imaging tests. Spirometry remains the gold standard for diagnosing ventilatory defects (2). It can detect asymptomatic cases with obstructive ventilatory defects as well as cases of impaired pulmonary function, even in the absence of obstructive ventilatory defects (7–11). Conversely, spirometry is usually performed in symptomatic patients (12), low uptake compared to that in chest radiography is the major problem of spirometry in preventive medicine. Moreover, participants must cooperate during the test and breathe with effort to obtain accurate results. Low throughput is an additional issue. Throughput is further limited in cases that require infection control measures. Thus, spirometry must be encouraged, and alternative tests with good throughput must be developed to overcome the challenges in performing PFTs during clinical examinations.

Imaging tests are associated with high throughput and a relatively high screening uptake rate. Chest radiographs (CXRs) remain the first choice of imaging test for cardiopulmonary screening and are commonly acquired during routine primary care, including health checkups. The CXR can visually identify morphological abnormalities in the lungs and other thoracic regions and can detect various diseases, for example, pneumonia and lung cancer. If the CXR shows abnormal findings related to pulmonary function, such as emphysema in COPD, this can be detected without spirometry. However, it is difficult to detect lesions that cause abnormal pulmonary function at an early stage with CXR, and, therefore, it is generally not used to assess pulmonary function. Thus, spirometry and CXR are complementary and have advantages and disadvantages. If cases with functional abnormalities can be detected in CXR without detectable image findings, it may lead to the creation of health-promoting opportunities for patients. Hence, it would be clinically useful if pulmonary function could be accurately obtained from the CXR.

Previous studies have estimated pulmonary function using the shape of the rib cage on CXRs acquired during static imaging (13–16). Similarly, studies have investigated the relationship between image characteristics and pulmonary function on dynamic chest X-ray radiographs (DCRs) acquired during dynamic imaging (17, 18). Pulmonary function has been estimated using image characteristics measured from landmarks in the images and regression models or equations; however, the accuracy of the estimated values was limited as the correlation between image features and lung function was not high. Furthermore, it requires manual measurement of image characteristics, a labor-intensive task, and may lead to errors. Machine learning has resulted in breakthroughs in medical image analysis in recent years, and several studies have used general image recognition models in medical image analysis and the estimation of functional parameters and other information from images (19). Sogancioglu et al. (20) reported the use of artificial intelligence (AI) for the estimation of the lung volume from pseudo-CXRs calculated from CT images. However, the estimated lung volumes were calculated from CT image data and not pulmonary function values. Schroeder et al. (21) reported the estimation of the % predFEV_1_ and FEV_1_/FVC as PFT values from bidirectional CXR pairs using deep learning. The study used two-view CXRs including imaging findings for estimation, not frontal CXRs alone. It was not clear whether pulmonary function impairment could be estimated from CXRs without imaging findings. Health checkups are performed routinely under the national system in Japan, and almost all adults undergo CXR screening. However, CXR screening is not always performed bidirectionally. It is important to determine whether accurate pulmonary function values can be obtained from frontal CXR images to develop an AI system for estimating pulmonary function from CXRs that can be used during medical examinations worldwide, including in developing countries.

Therefore, this study aimed to estimate the spirometry measurements from single frontal CXR without image findings using a general image recognition model and evaluate the precision of the estimation.

## 2 Materials and methods

This study was conducted after receiving approval for the use of medical data obtained during medical examinations from the Institutional Review Board of the Niigata University of Health and Welfare and the data-providing institutions (Approval number: 18952-221124).

### 2.1 Materials

#### 2.1.1 Data

**Figure 1:**
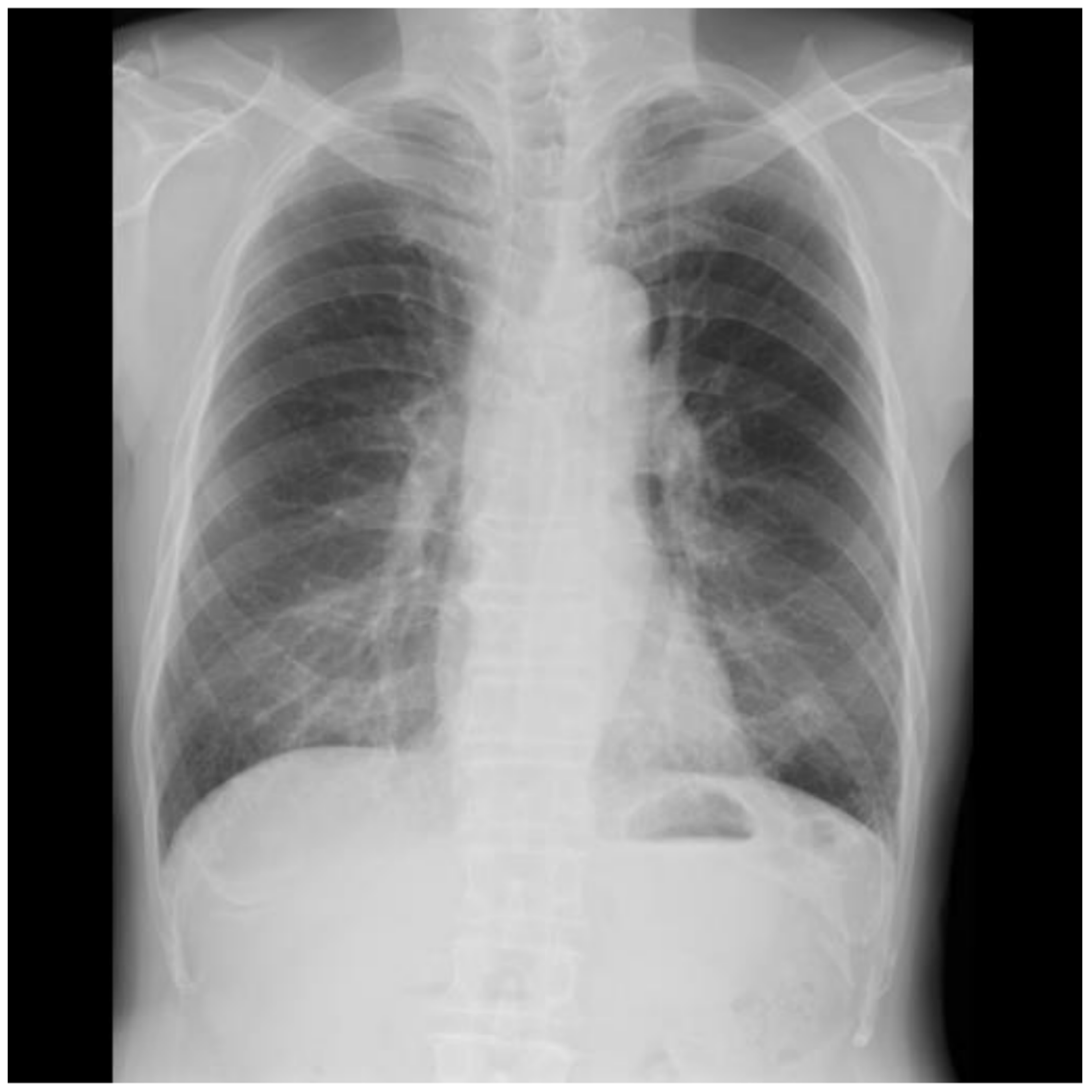
Sample chest radiographs used in this study. The original images were down-sampled and zero-padded to a 512 × 512 matrix with the aspect ratio preserved. Additionally, they were resampled to 224 × 224 and used as input.

**Figure 2:**
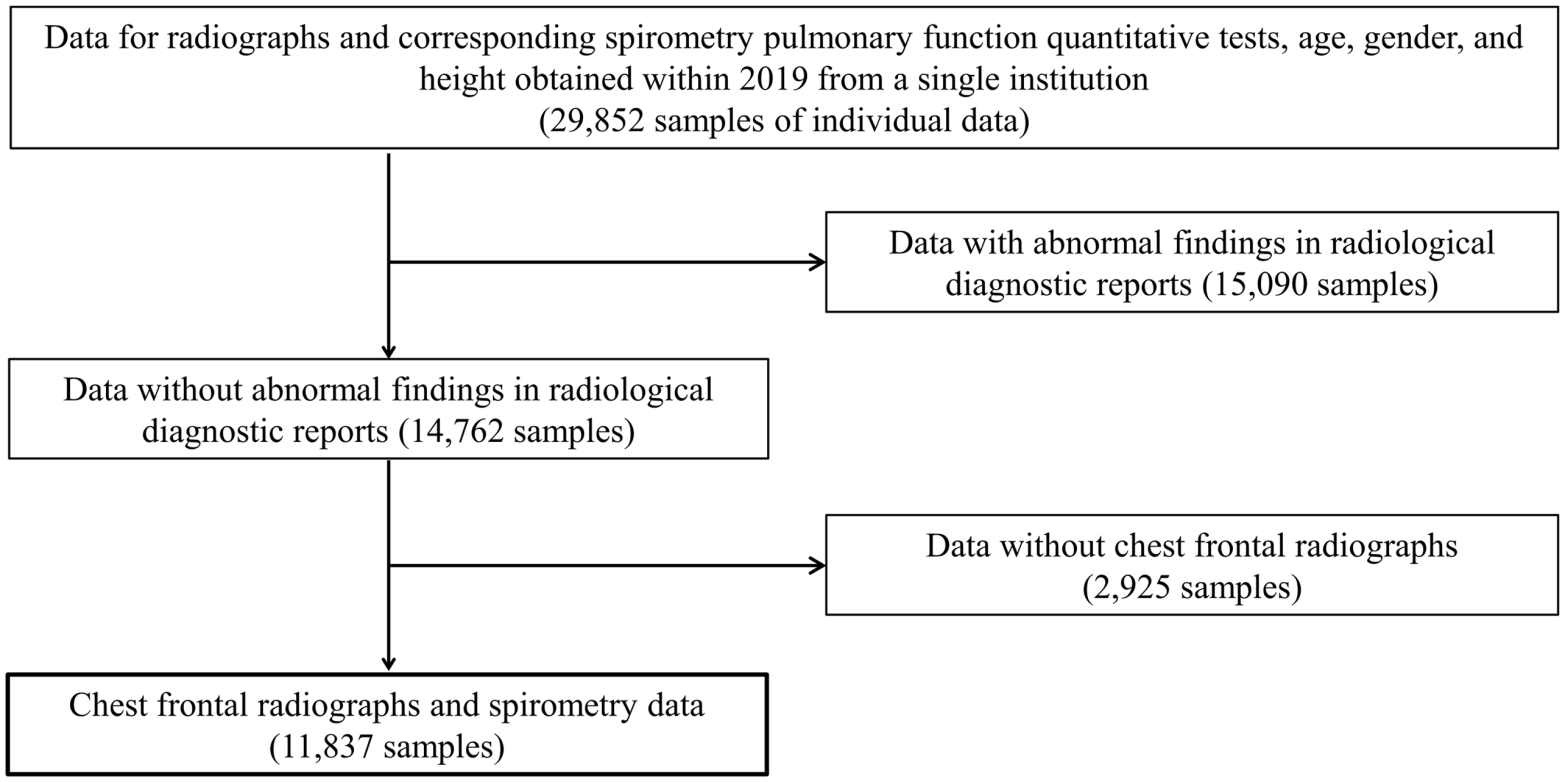
The inclusion and exclusion criteria for data acquisition. Only frontal chest radiographs and spirometry data obtained at a single institution with no abnormal findings on diagnostic reports and no history of undergoing radiography and spirometry on the same day were used.

Frontal CXRs acquired at a single institution in Japan for 2019 were used in this study. The CXR images in 8-bit PNG format were used. Figure 1 shows a representative CXR. The FVC, FEV_1_ and FEV_1_/FVC values obtained via forced vital capacity testing were used as the pre-bronchodilator spirometry data, as described in multiple COPD studies (22–24). Figure 2 presents the inclusion and exclusion criteria for the CXR and PFT data. The dataset used in this study are cases with no image findings noted in the radiology reports of the screening CXR. Cases with any abnormal findings such as lung opacities, lung cancer or other pulmonary disease, pleural lesions, cardiovascular lesions, musculoskeletal lesions, tracheal abnormalities, postoperative and supported devices were excluded. The CXRs in the dataset does not include any image findings noted, including inactive findings. The CXR data and corresponding PFT data were extracted from only one sample per participant. A total of 11,837 data samples, including the corresponding heights, sexes, and ages, were included in the PFT data; there were no missing data values. Table 1 presents the demographic characteristics of the datasets. A total of 9,469, 1,184, and 1,184 samples were used for the training, validation, and test of the deep learning network to ensure that the data ratio was maintained at 8:1:1.

**Table 1:** Demographic characteristics and pulmonary function indices of datasets.

		Training	Validation	Test	Overall
Participant	*n*	9,469	1,184	1,184	11,837
Sex	Female, *n* (%)	3,325 (35.1%)	425 (35.9%)	422 (35.6%)	4,172 (35.2%)
	Male, *n* (%)	6,144 (64.9%)	759 (64.1%)	762 (64.4%)	7,665 (64.8%)
Age (year)		50 [43–58]	50 [43–58]	50 [43–58]	50 [43–58]
Height (cm)		167.3 [160.5–172.8]	166.8 [160.2–172.6]	167.3 [160.1–173.0]	167.3 [160.4–172.8]
FVC (mL)		3,600 [2,980–4,210]	3,680 [3,038–4,320]	3,610 [2,960–4,180]	3,600 [2,980–4,210]
FEV1 (mL)		2,880 [2,410–3,400]	3,585 [2,978–4,240]	2,900 [2,398–3,413]	2,880 [2,410–3,400]
FEV1/FVC (%)		81.1 [77.0–84.8]	81 [76.8–84.8]	81.1 [77.0–84.7]	81.1 [77.0–84.8]
predFVC (mL)		3,890 [3,010–4,310]	3,880 [2,980–4,320]	3,885 [3,000–4,330]	3,890 [3,010–4,310]
predFEV1 (mL)		3,260 [2,530–3,700]	3,250 [2,498–3,710]	3,245 [2,520–3,710]	3,260 [2,520–3,700]
% FVC		97.8 [89.9–105.6]	97.8 [89.3–106.2]	97.3 [89.8–105.4]	97.6 [89.5–105.8]
% FEV1		97.7 [89.5–105.8]	93.8 [85.0–101.9]	93.4 [85.9–101.3]	93.6 [85.4–101.9]
% FEV1 category, *n* (%)	>80%	10,281 (86.9%)	1,020 (86.1%)	1,027 (86.7%)	10,281 (86.9%)
	50%–79%	1,528 (12.9%)	161 (13.6%)	154 (13%)	1,528 (12.9%)
	30%–49%	24 (0.2%)	1 (0.1%)	3 (0.3%)	24 (0.2%)
	<30%	4 (0%)	2 (0.2%)	0 (0%)	4 (0%)

Age, height, FVC, FEV_1_, FEV_1_/FVC, predFVC, predFEV1, % FVC, % FEV_1_ are shown as median [25–75th percentile values]. FVC, forced vital capacity; FEV_1_, forced expiratory volume in 1 s; predFVC, predicted FVC; pred FEV_1_, predicted FEV_1_; % FVC, percent-predicted FVC; % FEV_1_, percent-predicted FEV_1_.

#### 2.1.2 Experimental environment

MATLAB 2022a (MathWorks, Inc.) was used to implement the framework for performing the deep learning operations. Image processing and deep learning computations were performed using MATLAB in this study.

### 2.2 Methods

#### 2.2.1 Network training and evaluation

In addition to the pre-training data from the ImageNet classification task, ResNet-18, ResNet-50, ResNet-101, DenseNet-201, and Inception-ResNet-V2, which are publicly available in the MATLAB add-in library, were used as the initial weights (25, 26). The fully connected layers closest to the output layer of each network were replaced with a new layer with an output class of one. The training conditions were as follows: optimization method, Adam; loss function root mean square error; batch size, 32–256 (variable); initial learning rate, 1 × 10^−5^; maximum number of epochs, 50; image data augmentation, ±5° random rotation/random horizontal flip/±5% random scaling. The batch size was varied for each network type and then optimized. The network weights were updated using the training dataset, and the network performance at each epoch was displayed using the validation dataset. The weights in the epoch with the lowest loss for the validation dataset were saved to complete the learning. Network training and estimation were performed separately for FVC, FEV_1_ and FEV_1_/FVC.

#### 2.2.2 Evaluation

CXRs from the test dataset and the FVC or FEV_1_ estimations were the input and output of the network, respectively. The mean average percentage error (MAPE) and Pearson’s correlation coefficient (*r*) between the reference measured values and network-estimated values were used as the evaluation indices. Bland–Altman analysis (27) was performed using the reference measured value and the error between the estimated value and the measured value. The estimated value and the measured value were considered to be variables that could be treated equally if >95% of the evaluation data were included in the limits of agreement (LOA) at mean ± 1.96 SD.

## 3 Results

**Figure 3:**
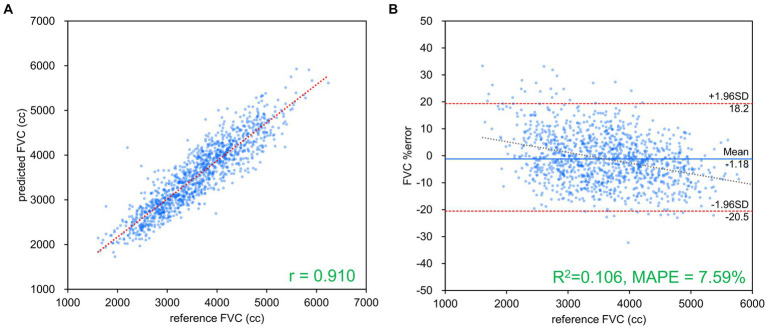
FVC estimation results using Inception-ResNet-V2. **(A)** Comparison of measured and estimated values. **(B)** Bland–Altman-like plot presenting the measured value-estimated error rate relationship. The correlation coefficient and error rate were the best among the networks used, with 96.1% of the data within the limits of agreement (mean ± 1.96 SD), confirming agreement between spirometry measurements and AI estimation using chest radiography. FVC, forced vital capacity.

**Figure 4:**
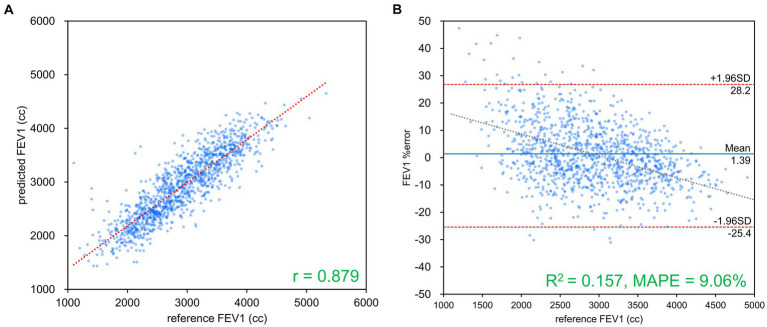
FEV_1_ estimation results using Inception-ResNet-V2. **(A)** Comparison of measured and estimated values. **(B)** Bland–Altman-like plot representing the measured value-estimated error rate relationship. The correlation coefficient and error rate were the best among the networks used, with 97.6% of the data within the limits of agreement (mean ± 1.96 SD), confirming agreement between spirometry measurements and AI estimation using chest radiography. FEV_1_, forced expiratory volume in 1 s.

**Figure 5:**
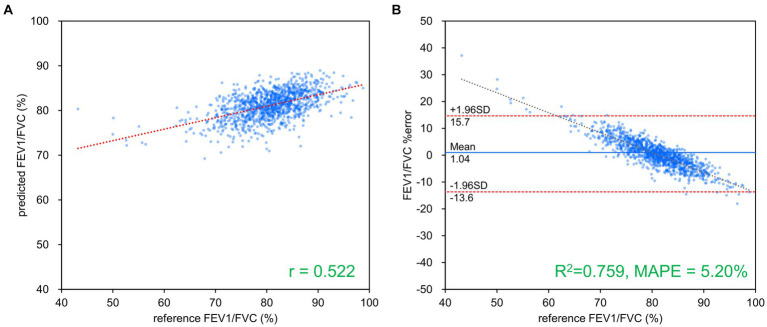
FEV_1_/FVC estimation results using ResNet-101. **(A)** Comparison of measured and estimated values. **(B)** Bland–Altman-like plot representing the measured value-estimated error rate relationship. The correlation between the estimated and measured values was moderate, while the error rate was low at about 5%. FVC, forced vital capacity; FEV_1_, forced expiratory volume in 1 s.

Table 2 presents the results of FVC, FEV_1_ and FEV_1_/FVC estimations for each network. FVC and FEV_1_ estimates showed strong positive correlations with both networks. Inception-ResNet-V2, which had the largest number of parameters, achieved the best MAPE and correlation coefficients for FVC and FEV_1_. The MAPE and correlation coefficients for FVC estimation were 7.585–8.246 and 0.903–0.910, respectively. The MAPE and correlation coefficients for FEV_1_ estimation were 9.055–9.442 and 0.865–0.879, respectively. The MAPE and correlation coefficients for FVC estimation were superior to those of FEV_1_ estimation, regardless of the network type used. Figure 3 presents the results of the comparison between the FVC estimation results of the Inception-ResNet-V2 network, which yielded the lowest MAPE and the highest correlation coefficient, and the reference. Figure 4 presents the results of the comparison between the FEV_1_ estimation results and the reference. The 95% confidence interval for the mean error rate of FVC estimation (Figure 3B) ranged between −1.741% and −0.615% in the Bland–Altman plot. The slope of the coefficient for the determination of the % error-reference of an approximately straight line, *R*^2^ = 0.106, was not significant. The agreement between the estimated and measured FVC values was confirmed, as 96.1% of the data were included within the LOA. The 95% confidence interval for the mean percentage error of FEV_1_ estimation ranged between 0.606% and 2.164% in the Bland–Altman plot (Figure 4B). The slope of the coefficient for the determination for the % error-reference of an approximately straight line, *R*^2^ = 0.157, was not significant. The agreement between the FEV_1_ estimates and measured values was confirmed, as 97.6% of the data were included within the LOA. Figure 5 presents the results of the deep learning network with the best correlation coefficient and MAPE for estimating FEV_1_/FVC. The MAPE was acceptable at 5.20%, whereas the correlation was moderate at *r* = 0.522. The correlation between FEV_1_/FVC estimates and measured values was weaker than those observed for the estimation of FVC and FEV_1_. The 95% confidence interval for the mean error rate of FVC estimation (Figure 5B) ranged between −221213.6% and 15.7% in the Bland–Altman plot. The slope of the coefficient for the determination for the % error-reference of an approximately straight line, *R*^2^ = 0.759, was significant. The agreement between the FEV_1_/FVC estimates and measured values was confirmed, as 96.8% of the data were included within the LOA.

**Table 2:** Comparison of estimation performance of the network for each pulmonary function indices.

Network	FVC		FEV_1_		FEV_1_/FVC	
	MAPE	*r*	MAPE	*r*	MAPE	*r*
ResNet-18	7.995	0.903	9.442	0.865	5.344	0.492
ResNet-50	8.083	0.907	9.063	0.871	5.885	0.465
ResNet-101	8.206	0.909	9.328	0.866	5.210	0.522
DenseNet-201	8.101	0.909	9.249	0.876	5.565	0.466
Inception-ResNet-V2	7.585	0.910	9.055	0.879	5.674	0.512

FVC, forced vital capacity; FEV_1_, forced expiratory volume in 1 s.

## 4 Discussion

A typical deep learning network was used to estimate the FVC, FEV_1_ and FEV_1_/FVC values from a frontal CXR via spirometry in this study. Strong positive correlations were observed between the estimated FVC and FEV_1_ values and the corresponding measured values. The MAPE was low (<10%) for FVC, FEV_1_ and FEV_1_/FVC estimations. The Bland–Altman analysis revealed good agreement between the estimated and measured values for FVC and FEV_1_. Thus, the findings of this study indicate that frontal CXRs contain information related to pulmonary function and that AI estimation performed using frontal CXRs can estimate spirometry-measured values FVC and FEV_1_ with high accuracy.

The pulmonary function parameters to be estimated in this study were FVC and FEV_1_, which are expiratory volumes exhaled during forced breathing with no time limit. FVC is the total expiratory volume exhaled during forced breathing without any time limit, whereas FEV_1_ is the expiratory volume exhaled during the first second of forced breathing. Thus, FEV_1_ can be considered a part of FVC, where FEV_1_ is the flow velocity. FEV_1_, a highly sensitive indicator of decreased ventilatory capacity, is decreased in patients with obstructive ventilatory defects owing to air trapping caused by damaged alveoli, which increases the peripheral airway resistance and limits the expiratory volume that can be exhaled in a short period of time (28–30). This decrease in FEV_1_ is particularly significant in patients with progressive COPD; however, it can also be observed in the pre-COPD stage and early stages of COPD, wherein the decrease in ventilation capacity is less evident (31). Specific findings are observed on the CXRs of patients with severe COPD; however, such findings are not observed in patients with early-stage COPD. Therefore, it is reasonable to assume that the accuracy of FEV_1_ estimation is relatively inferior to that of FVC estimation, an index that varies more frequently among patients. The correlation of the estimated FEV_1_/FVC and those of measurements was weaker than those observed for the case of estimation of FVC and FEV_1_. This may be attributed to the individual variability of FVC and FEV_1_, which makes the FEV_1_/FVC value a more complex predictor.

**Figure 6:**
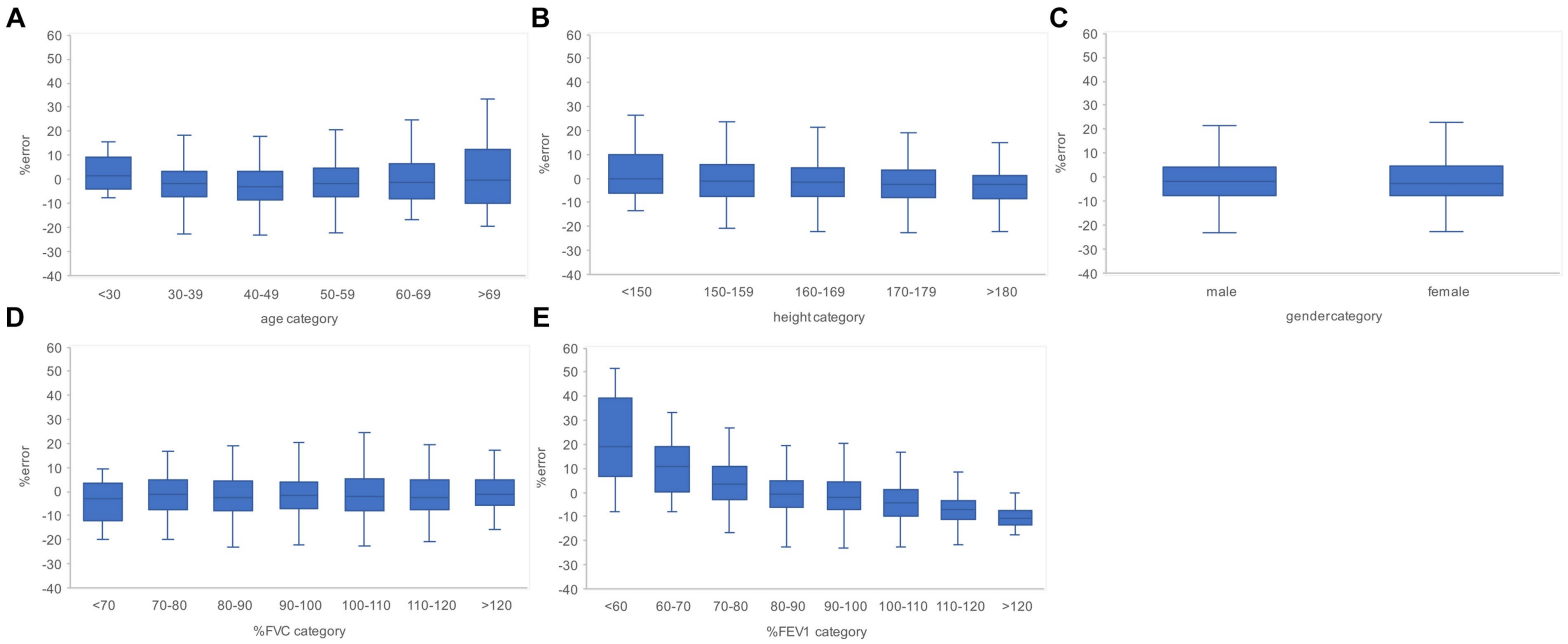
Relationship between the subgroups and FVC estimation error rates in the evaluation data. **(A)** Percentage error by age category. **(B)** Percentage error by height category. **(C)** Percentage error by gender. **(D)** Percentage error per % FVC category. **(E)** Percentage error by % FEV_1_ category. The higher the age category and the lower the % FEV_1_ category, the larger the variance of the percentage error tended to be. FVC, forced vital capacity; FEV_1_, forced expiratory volume in 1 s.

**Figure 7:**
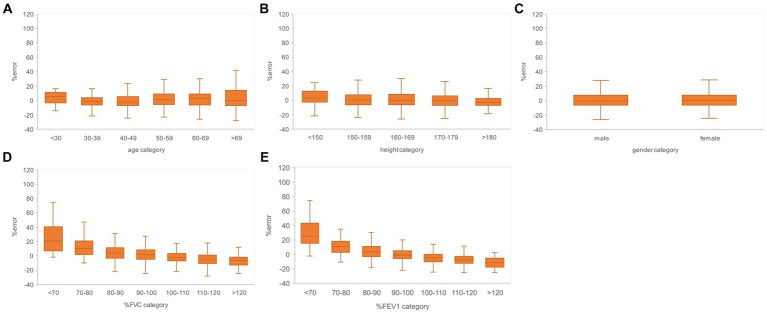
Relationship between the subgroups and FEV_1_ estimation error rates in the evaluation data. **(A)** Percentage error by age category. **(B)** Percentage error by height category. **(C)** Percentage error by gender. **(D)** Percentage error per % FVC category. **(E)** Percentage error by % FEV_1_ category. The variance of the percent error tended to be larger for the higher age categories and for the lower % FVC and % FEV_1_ categories. FVC, forced vital capacity; FEV_1_, forced expiratory volume in 1 s.

**Figure 8:**
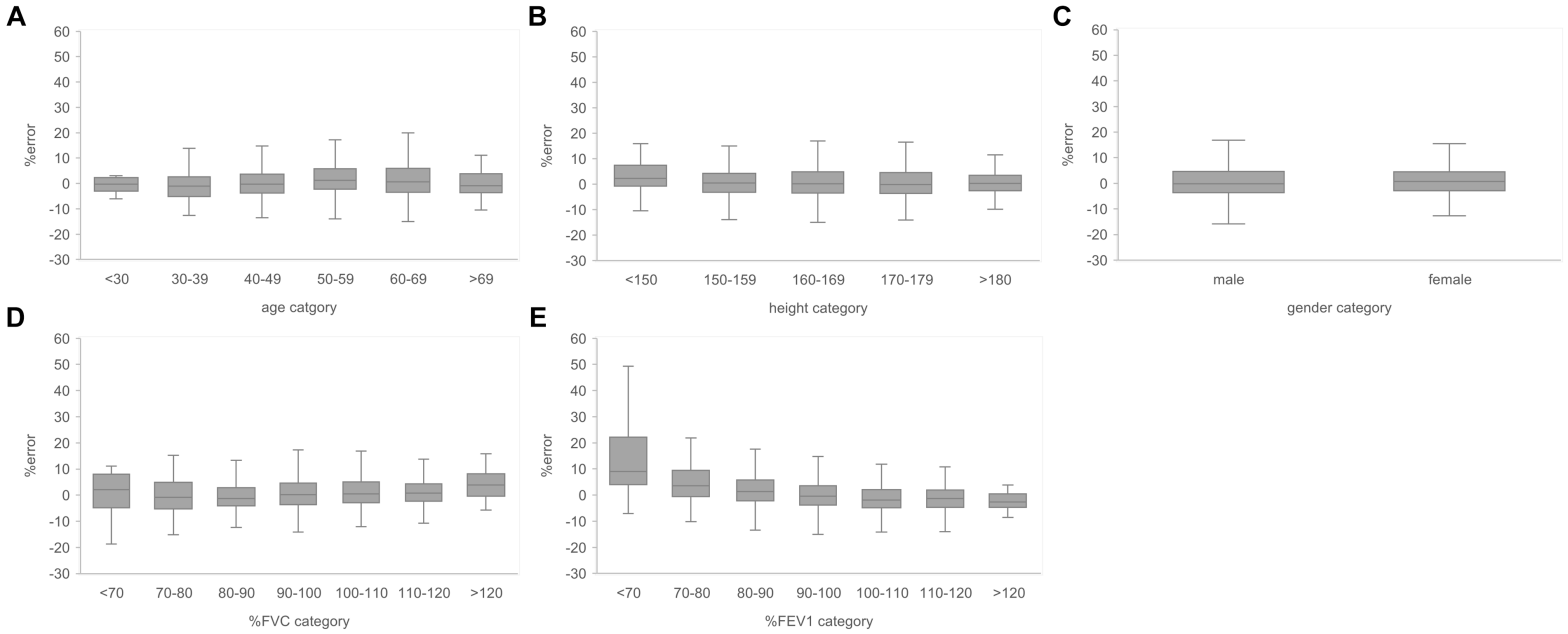
Relationship between the subgroups and FEV1/FVC estimation error rates in the evaluation data. **(A)** Percentage error by age category. **(B)** Percentage error by height category. **(C)** Percentage error by gender. **(D)** Percentage error per % FVC category. **(E)** Percentage error by % FEV1 category. The variance of the percent error tended to be larger for the lower % FEV1 categories. FVC, forced vital capacity; FEV_1_, forced expiratory volume in 1 s.

Subgroups were created based on the age, height, sex, % FVC, and % FEV_1_ related to the estimation error to increase the robustness of the performance of the AI estimation method used in this study. Age, height, and sex are the information used to determine the % FVC and % FEV_1_ in spirometry. The % FVC and % FEV_1_ are relative to the predicted FVC and FEV_1_ values, respectively, which are standard values for the same age, height, and sex expressed as percentages. Thus, % FVC and % FEV_1_ are indicators of a participant’s pulmonary function relative to the standard population. Each subgroup, except for the subgroup created on the basis of sex, was divided into categories, and the error rates for each category were compared. The categories for each subgroup were as follows: age category, <30 years, 30–49 years, 50–59 years, 60–69 years, and >70 years; height category, <150 cm, 150–160 cm, 160–170 cm, 170–180 cm, and >180 cm; sex category, male and female; % FVC category, <70, 70–80, 80–90, 90–100, 100–110, 110–120, and >120, % FEV_1_ category <70, 70–80, 80–90, 90–100, 100–110, 110–120, and >120. Differences in the distributions of error rates among categories were tested using the Kruskal–Wallis method (significance level *p* < 0.05) and multiple comparisons. Figure 6 presents the distributions of error rates in the FVC estimation according to the subgroup and category. The distribution of error rates tended to widen with increasing age in the age category (Figure 6A); however, multiple comparisons performed using the Kruskal–Wallis test revealed no statistically significant differences among the categories. There were no trends or significant differences in height, sex, or % FVC subgroups (Figure 6B-D). Significant differences were observed in the distribution between the categories with low % FEV_1_ and the other categories in the % FEV_1_ subgroup (*p* < 0.001). Figure 7 presents the distributions of error rates in the FEV_1_ estimation according to the subgroup and category. No significant differences were observed between the categories in terms of age, height, or sex. Thus, the findings suggest that robust performance was obtained without error bias for age, height, and sex. Significant differences were observed in the errors between categories with low % FVC values and several other categories in the % FVC subgroup. Multiple comparisons revealed the relationship between each % FEV_1_ category and the FEV_1_ estimation error rate (Figure 7E). Significantly different mean ranks were observed between all categories except between categories 100–110 and 110–120 and between 110–120 and >120 in the % FEV_1_ subgroup (*p* < 0.001). Figure 8 presents the distributions of error rates in the FEV_1_/FVC estimation according to the subgroup and category. No significant differences were observed between the categories in terms of height and sex. Thus, the findings suggest that a robust performance was obtained without error bias for height and sex. Multiple comparisons revealed the relationship between each % FEV_1_ category and the FEV_1_/FVC estimation error rate (Figure 8E). Significantly different mean ranks were observed between categories between categories 30–39 years and 50–59 years in the age subgroup (*p* < 0.01) and between categories between categories 80–90 and >120 in the % FVC subgroup. Additionally, in the % FEV_1_ subgroup, significantly different mean ranks were observed between all categories (*p* < 0.05), except between categories <70 and 70–80; 70–80 and 80–90; 80–90 and 100–110; 100–110 and 110–120 and >120; 110–120 and >120. Table 3 presents the number of data points and error rates for each subgroup and category. The median error tended to be more positively biased for categories with a lower % FEV_1_ in the % FEV_1_ subgroup. It is suspected that the low % FVC and low % FEV_1_ categories had small samples and that the characteristics of % FVC and % FEV_1_ might not have been learned sufficiently. However, the error rate did not increase significantly for the other age and height categories with a lesser amount of data. Therefore, the results of this study do not exclude the possibility that the relationship between lower % FVC and % FEV_1_ and imaging features has not been sufficiently trained by network. Future studies should increase the number of samples with low % FVC and % FEV_1_ during training and validate the robustness of the % FVC and % FEV_1_ subgroups.

**Table 3:** Number of test data and percentage error of AI estimation according to the age, height, sex, % FVC and % FEV_1_.

Subgroup	Category	*n*	Absolute percentage error (mean ± SD)		
			FVC	FEV_1_	FEV_1_/FVC
Age (year)	<30	12	6.43 ± 4.98	7.96 ± 5.30	2.96 ± 3.30
	30–39	135	6.86 ± 5.56	7.25 ± 6.18	4.82 ± 3.63
	40–49	420	7.11 ± 8.44	7.84 ± 9.76	4.70 ± 6.45
	50–59	382	7.50 ± 7.40	9.57 ± 13.12	5.23 ± 4.96
	60–69	181	8.43 ± 6.87	11.07 ± 13.03	6.58 ± 8.64
	>69	54	11.00 ± 7.38	12.76 ± 12.36	5.74 ± 6.07
Height (cm)	<150	31	7.97 ± 7.00	10.21 ± 9.6	5.37 ± 4.36
	150–159	264	7.94 ± 6.48	8.94 ± 8.13	4.67 ± 3.72
	160–169	437	7.66 ± 7.23	9.86 ± 13.60	5.83 ± 7.22
	170–179	399	7.33 ± 5.45	8.37 ± 7.13	4.94 ± 4.05
	>180	53	6.76 ± 5.86	7.33 ± 9.57	4.64 ± 5.53
Sex	Male	762	7.46 ± 6.63	9.10 ± 11.46	5.35 ± 5.83
	Female	422	7.79 ± 6.07	8.96 ± 7.93	4.94 ± 4.73
% FVC	<70	16	7.38 ± 5.88	38.24 ± 52.84	9.77 ± 15.57
	70–80	59	7.29 ± 9.18	13.12 ± 12.57	5.63 ± 6.98
	80–90	227	7.41 ± 9.21	9.36 ± 11.44	5.3 ± 7.18
	90–100	386	7.33 ± 10.15	8.9 ± 12.54	5.42 ± 8.76
	100–110	316	7.9 ± 9.64	6.96 ± 8.69	4.85 ± 6.03
	110–120	143	8.02 ± 10.99	8.63 ± 8.96	4.58 ± 5.98
	>120	37	7.3 ± 10.35	9.13 ± 8.22	5.12 ± 5.29
% FEV1	<70	40	16.3 ± 14.61	35.36 ± 24.07	16.14 ± 17.98
	70–80	117	8.29 ± 9.84	12.37 ± 10.46	6.72 ± 7.46
	80–90	292	7.01 ± 9.1	8.43 ± 9.93	4.98 ± 6.28
	90–100	394	6.71 ± 8.24	6.69 ± 8.36	4.64 ± 5.86
	100–110	223	7.43 ± 7.92	7.25 ± 7.69	4.41 ± 5.33
	110–120	94	8.22 ± 6.7	9.1 ± 8.03	3.99 ± 4.88
	>120	24	9.73 ± 5.02	11.82 ± 7.1	3.73 ± 4.01

FVC, forced vital capacity; FEV_1_, forced expiratory volume in 1 s; % FVC, percent-predicted FVC; % FEV_1_, percent-predicted FEV_1_.

Previous studies investigating the relationship between CXRs and pulmonary function manually extracted image characteristics from dynamic CXRs to investigated their correlations with pulmonary function. Hino et al. (17) and Hida et al. (18) investigated the correlation between image characteristics and pulmonary function values on dynamic CXR. In the study by Hino et al. (17), the highest correlation coefficient was between lung field area and FEV_1_ at the maximal inspiratory position with effort breathing in DCR was the highest (right *r* = 0.59, left *r* = 0.62). The study by Hida et al. (18) also showed the highest correlation between whole excursion of the diaphragm and FEV_1_ in DCR, although the correlation was weak (right *r* = 0.27, left *r* = 0.38). While previous studies have reported a moderate correlation between FEV_1_ and lung field area based on DCR image measurements, this study used deep learning networks to automatically extract and select image characteristics from static CXRs and revealed a strong positive correlation (*r* = 0.879) between CXRs and FEV_1_. The findings of this study suggest that pulmonary function can be estimated accurately from static images using deep learning networks, resulting in a significant improvement in accuracy. In a previous study in which pulmonary function was estimated from CXR using machine learning, Schroeder et al. (21) estimated FEV_1_/FVC using bidirectional CXR pair and obtained *R*^2^ = 0.415 (conversion *r* = 0.644), which is a moderately positive correlation. In this study, only frontal CXR was used to estimate FEV1/FVC. An *R*^2^ = 0.272 (*r* = 0.522) was obtained, indicating a moderate positive correlation. The absence of lateral CXR in this study is expected to have resulted in the deep learning network extracting less information compared to if bidirectional CXR pairs were utilized, leading to lower estimation performance.

Among pathologies with obstructive ventilation defects, COPD is the most common chronic respiratory disease worldwide, with approximately 174 million affected individuals (32). COPD is an irreversible pathology; thus, it is important to detect and initiate treatment prior to its progression. However, the symptoms of COPD only become apparent as the disease reaches advanced stages. Moreover, it is difficult to detect COPD early using CXRs. Therefore, detecting and initiating treatment at the earliest possible stage for patients with COPD who are asymptomatic has become an important public health issue worldwide. In this study, the FVC and FEV_1_ values measured using spirometry could be estimated with an average accuracy of >90% using only frontal CXRs, which are the most commonly acquired images in imaging tests, in this study. The method used in this study provides spirometry estimates without any additional burden to the CXR examinee. In the future, if the robustness of the estimation performance to the characteristics of the data is sufficiently verified, estimation of pulmonary function using CXR could be used as an adjunct to spirometry in individuals with low estimated pulmonary function or as an alternative to pulmonary function measurement. Chest radiography (screening CXR) is a low-cost and relatively widespread cancer screening method that can be used as an alternative for the COPD risk assessment. The findings of this study suggest that FVC and FEV_1_ could be estimated with an average accuracy of >90% and >87% for participants with % FEV_1_ of >80% and >70%, respectively. Thus, the network developed in this study could be used as an alternative for COPD risk assessment in patients with mildly impaired pulmonary function and for the control of the pre-COPD group.

This study has some limitations. Only cases with no abnormal findings in the CXR report were used to eliminate the influence of abnormal findings on the estimation of pulmonary function by image features of abnormal findings. Another reason is that it is significant for use in estimating pulmonary function is CXR without abnormal findings related to abnormalities in pulmonary function. However, the available training data can be expected to increase and a higher network performance can be achieved if the pulmonary function can be estimated accurately, even in cases with abnormal findings. The results of this study did not exclude the possibility of inferior estimation performance by deep learning for cases with lower % FVC and % FEV_1_. To validate and further generalize the findings of this study, it will be necessary to train a larger number of samples with low % FVC and % FEV_1_ and to perform external validation using data from another facility. Only ImageNet-pretrained networks publicly available in MATLAB and general deep-learning networks were used in this study. Depending on the samples and networks used, a larger network scale had greater correlation coefficient and MAPE. Thus, it is possible that larger deep learning networks can be used to develop pulmonary function estimation networks with higher performance.

## 5 Conclusion

Pulmonary function values measured using spirometry were estimated from the corresponding frontal CXRs using a general deep learning network. FVC, FEV_1_ and FEV_1_/FVC were estimated with an average accuracy of >90%. The pulmonary function estimation network developed in this study may be a useful method for pulmonary function screening or a potential substitute for spirometry.

## Data availability statement

The raw data supporting the conclusions of this article will be made available by the authors, without undue reservation.

## Ethics statement

The studies involving humans were approved by Ethics Committee of Niigata University of Health and Welfare. The studies were conducted in accordance with the local legislation and institutional requirements. The ethics committee/institutional review board waived the requirement of written informed consent for participation from the participants or the participants’ legal guardians/next of kin because this study was conducted using only anonymously processed information provided by Konica Minolta, Inc.

## Author contributions

AY: Conceptualization, Data curation, Formal analysis, Investigation, Methodology, Software, Validation, Writing – original draft, Writing – review & editing. CK: Writing – review & editing. HF: Resources, Writing – review & editing. KO: Resources, Writing – review & editing. SKo: Writing – review & editing. IS: Writing – review & editing. SKa: Conceptualization, Methodology, Project administration, Supervision, Writing – review & editing.
